# Bleeding Disorders in Primary Fibrinolysis

**DOI:** 10.3390/ijms22137027

**Published:** 2021-06-29

**Authors:** Massimo Franchini, Marco Zaffanello, Pier Mannuccio Mannucci

**Affiliations:** 1 Department of Transfusion Medicine and Hematology, Carlo Poma Hospital, 46100 Mantova, Italy; 2 Department of Surgical Sciences, Dentistry, Gynecology and Pediatrics, University of Verona, 37126 Verona, Italy; marco.zaffanello@univr.it; 3 Angelo Bianchi Bonomi Hemophilia and Thrombosis Center, Fondazione IRCCS Ca’ Granda-Ospedale Maggiore Policlinico and University of Milan, 20122 Milan, Italy; piermannuccio.mannucci@policlinico.mi.it

**Keywords:** primary hyperfibrinolysis, bleeding, hemorrhage, inherited, acquired

## Abstract

Fibrinolysis is a complex enzymatic process aimed at dissolving blood clots to prevent vascular occlusions. The fibrinolytic system is composed of a number of cofactors that, by regulating fibrin degradation, maintain the hemostatic balance. A dysregulation of fibrinolysis is associated with various pathological processes that result, depending on the type of abnormality, in prothrombotic or hemorrhagic states. This narrative review is focused on the congenital and acquired disorders of primary fibrinolysis in both adults and children characterized by a hyperfibrinolytic state with a bleeding phenotype.

## 1. Introduction

**Figure 1:**
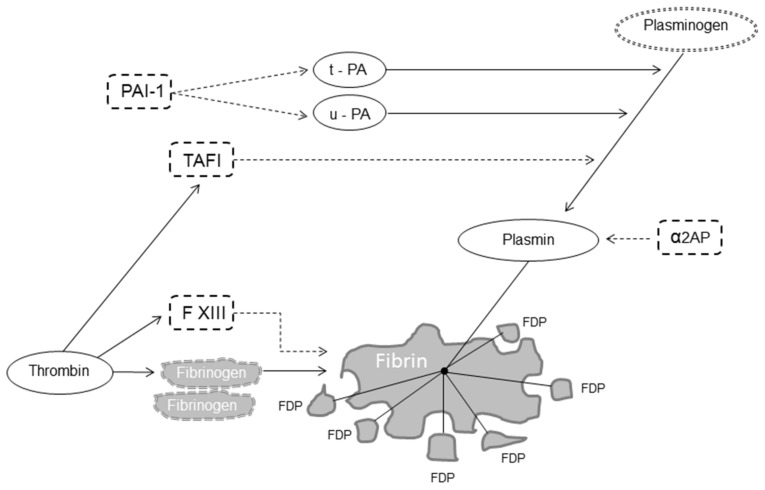
Fibrinolysis. Legend: dot arrow inhibits fibrinolysis; solid line favors fibrinolysis. Abbreviations: FXIII, factor XIII, t-PA, tissue plasminogen activator; u-PA, urokinase plasminogen activator; α2AP, α_2_-antiplasmin; PAI-1, plasminogen activator inhibitor 1; TAFI, thrombin-activatable fibrinolysis inhibitor; FDP, fibrin degradation products.

Fibrinolysis is a delicate and complex enzymatic process aimed at dissolving blood clots, thereby localizing and limiting clot formation [1,2,3]. Fibrinolysis is responsible for fibrin degradation and is modulated by proteases and protease inhibitors that, having the opposite effect, regulate the conversion of plasminogen to plasmin, the active enzyme that dissolves the fibrin clot into soluble fibrin degradation products (FDP) [4]. The fibrinolytic system thus includes pro-fibrinolytic (i.e., tissue plasminogen activator (t-PA) and urokinase plasminogen activator (u-PA)) and anti-fibrinolytic components (i.e., α2-antiplasmin, plasminogen activator inhibitor 1 (PAI-1) and thrombin-activatable fibrinolysis inhibitor (TAFI)) (Figure 1) [5].

While under physiological conditions, fibrinolysis (also called primary fibrinolysis) is a state of balance between these two opposite modulators; an abnormally reduced or excessive fibrinolysis disrupts the hemostatic balance and can lead to thrombosis or clinical bleeding, respectively [6]. Conditions causing a hypo- or hyper-fibrinolytic state are inherited, typically caused by a single molecular defect or more frequently acquired [6,7]. In this narrative review, we summarize the main pathogenetic, laboratory, clinical and therapeutic characteristics of inherited and acquired bleeding conditions associated with primary hyperfibrinolysis, which includes qualitative or quantitative abnormalities of proteins involved in the fibrinolytic process (see also Table 1).

## 2. Search Strategy

As a search literature strategy, the Medline, PubMed and Google Scholar electronic databases were searched for publications on bleeding disorders in primary fibrinolysis, without temporal limits, using English language as a restriction. The Medical Subject Heading and keywords used were: “abnormal fibrinolysis”, “bleeding disorders”, “inherited”, “acquired”, “hyperfibrinolysis”, “primary fibrinolysis”. We also screened the reference lists of the most relevant review articles for further studies not captured in our initial literature search. Search terms were also applied to abstracts from the most recent international congresses on hemostasis, thrombosis and hematology.

## 3. Inherited Bleeding Disorders of Primary Fibrinolysis

The inherited bleeding disorders of primary fibrinolysis include α_2_-antiplasmin deficiency, plasminogen activator inhibitor-1 deficiency and the Quebec platelet disorder.

### 3.1. α2-Antiplasmin Deficiency

The α_2_-plasmin inhibitor, a single-chain glycoprotein of 70 kDa synthesized by the liver, is the primary physiological inhibitor of plasmin [8]. Congenital α_2_-antiplasmin deficiency is a rare autosomal recessive condition (the gene is mapped to chromosome 17) characterized by clinical bleeding due to premature dissolution of hemostatic plugs, typically presenting as re-bleeding following trauma or invasive/surgical procedures [9,10]. α_2_-plasmin inhibits fibrinolysis by forming a stable inactive complex with plasmin by binding to the lysine-binding site of plasminogen and thus completely inhibiting plasminogen binding to fibrin, and by covalent binding to fibrin via activated factor XIII (FXIIIa) to prevent fibrinolysis by plasmin [1,11].

The diagnosis of α_2_-antiplasmin deficiency is difficult because coagulation screening tests are normal, but the euglobulin clot lysis time (ECLT) is short due to uninhibited plasmin activity. The functional and immunologic plasma assays of the inhibitor confirm the diagnosis [10]. Several mutations have been identified in the gene encoding α_2_-antiplasmin that cause two different types of α_2_-antiplasmin deficiency: a quantitative defect (type I), characterized by a similar decrease in both antigen and activity plasma levels, and a qualitative defect (type II), characterized by lower functional activity contrasting with normal antigen levels [10].

Case reports of both children and adults with congenital deficiency of α_2_-plasmin inhibitor [12,13,14,15,16] include a wide array of bleeding manifestations, such as umbilical cord bleeding, prolonged bleeding from wounds, epistaxis, gingival bleeding, subcutaneous and intramuscular hematomas, hematuria, hemarthroses and central nervous system bleeding. However, only homozygotes (presenting with α_2_-plasmin inhibitor levels < 10%) experience a significant bleeding tendency, usually severe and starting from childhood, while in heterozygotes (with levels between 20% and 50%), hemorrhagic events are reported to occur only after trauma, dental extractions or surgery [10]. The use of the antifibrinolytic agent tranexamic acid (TXA) is effective for preventing bleeding during or after invasive/surgical procedures and treating acute bleeding episodes. Fresh-frozen plasma (FFP) may be used as an alternative or an adjunct to antifibrinolytic therapy when an immediate increase of this fibrinolysis inhibitor is required [17]. Solvent–detergent treated plasma has lower α2-antiplasmin levels and thus is not recommended in the treatment of α2-antiplasmin deficiency [7].

### 3.2. Plasminogen Activator Inhibitor-1 Deficiency

PAI-1 is a single-chain glycoprotein of 52 kDa synthesized in the liver (the *PAI-1* gene is located at chromosome 7), which controls the proteolytic action of plasmin through inhibition of the plasminogen activators t-PA and u-PA [18,19,20]. Congenital PAI-1 deficiency, transmitted as an autosomal recessive trait, may be quantitative (decreased or absent protein production) or qualitative (dysfunctional PAI-1 synthesis with detectable protein but reduced or absent functional activity) [19]. As for α_2_-antiplasmin deficiency, the diagnosis of congenital PAI-1 deficiency is challenging because screening coagulation tests are normal. Although the ECLT is short and whole blood clotting assays such as thromboelastogram are abnormal, diagnosis is based on the results of antigenic (enzyme-linked immunosorbent assay, ELISA) and functional (chromogenic test) PAI-1 assays [19]. Congenital PAI-1 deficiency is an extremely rare bleeding disorder. While heterozygotes usually have no bleeding manifestations, PAI-1 deficiency in homozygotes appears to be a mild to moderate bleeding disorder [21,22,23,24,25]. These patients rarely have spontaneous bleeding but experience abnormal bleeding only following trauma or surgery. The bleeding tendency from congenital PAI-1 deficiency was also displayed in infancy [19] and children [24]. Menorrhagia and obstetric complications (miscarriages and preterm births) have been reported in PAI-1 deficient females [26]. Antifibrinolytic agents such as TXA are the mainstay of treatment of acute bleeding. The prophylactic administration of this drug is also very effective in preventing bleeding in patients undergoing surgery, especially oral and urogenital procedures. Persistent menorrhagia may be effectively managed with hormonal therapy and long-term prophylactic antifibrinolytic therapy if necessary. TXA is used at typical doses of 25 mg/kg body weight every 8 h. The duration of treatment for prevention and treating bleeds is usually 3–4 days for minor bleeds/surgeries and 5–7 days for major bleeds/surgeries. Desmopressin acetate should be avoided as it may induce endothelial secretion of plasminogen activator [7].

### 3.3. Quebec Platelet Disorder

The Quebec platelet syndrome is a rare autosomal dominant bleeding disorder associated with a mildly reduced platelet count and caused by overexpression of u-PA and its increased storage in platelet α-granules, leading to plasmin-mediated α-granule protein degradation, increased release of u-PA and hyperfibrinolysis [27,28,29]. The candidate gene *PLAU* (urokinase plasminogen activator) is mapped on chromosome 10 [30]. The laboratory characteristic is a marked increase of platelet content of the u-PA. From a clinical point of view, patients with the Quebec platelet syndrome typically present with delayed bleeding following trauma or surgical and dental procedures, but can also experience easy bruising, epistaxis, hematuria, menorrhagia and joint bleeds [31]. Sometimes bleeding may be severe and uncontrolled despite therapy. The mainstay of prevention and treatment of bleeding is based on antifibrinolytic agents, which should be avoided in patients with hematuria due to the risk of urinary tract clot formation leading to renal obstruction [28]. Prophylaxis with fibrinolytic inhibitors has also been successfully used during childbirth [31]. Platelet transfusions have been used but may not be beneficial in the case of severe bleeding [29].

## 4. Acquired Bleeding Disorders of Primary Fibrinolysis

The acquired bleeding disorders of primary fibrinolysis include liver cirrhosis, acute promyelocytic leukemia, severe trauma and post-partum hemorrhage.

### 4.1. Liver Cirrhosis

Severe liver disease is frequently complicated by clinically significant bleeding due to multiple causes, including endothelial dysfunction and thrombocytopenia caused by splenic sequestration, portal hypertension with the development of varices and a de-creased synthesis of coagulation factors and their inhibitors associated with impaired hepatic function [32,33,34].

A hyperfibrinolytic state is a common finding in liver cirrhosis and may [35,36,37] occur in up to 50% of patients with end-stage liver disease and is associated with mucocutaneous and gastrointestinal bleeding [38]. In this clinical setting, the fibrinolytic system is strongly activated by an increased endothelial release and decreased hepatic clearance of t-PA, decreased synthesis of TAFI, α_2_-plasmin inhibitor and PAI-1 [39,40,41,42]. In a study of 112 patients with liver cirrhosis with esophageal varices but without upper gastrointestinal bleeding followed for 3 years, elevated t-PA and D-dimer levels were the only markers predictive of bleeding [43]. The successful use of antifibrinolytic agents in patients with cirrhosis, particularly those with mucosal or gastrointestinal bleeding, further supports the mechanistic role of primary hyperfibrinolysis in this clinical setting [32].

Hyperfibrinolysis may also be responsible for cases of severe bleeding in patients undergoing orthotopic liver transplantation (OLT), who show accelerated fibrinolysis (especially during the anhepatic phase) associated with increased t-PA and reduced α_2_-plasmin inhibitor plasma levels [44]. In a double-blind, placebo-controlled randomized trial (RCT) on 137 patients undergoing OLT, Porte and colleagues [45] showed that the intraoperative use of the antifibrinolytic agent aprotinin significantly reduced blood-transfusion requirements. Moreover, a Cochrane review concluded that antifibrinolytic therapy helps to reduce blood loss and perioperative transfusion needs in this type of surgery [46].

### 4.2. Acute Promyelocytic Leukemia

Acute promyelocytic leukemia (APL) has peculiar molecular, morphological and clinical characteristics that render this type of acute myeloid leukemia unique [47]. A single genetic defect has been identified in APL, a translocation between chromosomes 15 and 17 resulting in the fusion of the promyelocytic leukemia protein gene (*PML*) and the retinoic acid receptor-α gene (*RARA*). The expressed PML-RAR-α fusion protein stops the differentiation of myeloid precursor cells and prolongs their survival, resulting in APL [48]. APL is characterized by an increased incidence of severe bleeding complications [47]. The hemostatic abnormalities in APL have the laboratory features of primary hyperfibrinolysis (i.e., elevated circulating levels of u-PA and t-PA and reduced levels of PAI-1, α2-antiplasmin and TAFI), and the responsible molecular mechanism has been elucidated in the last decade [49,50,51,52,53,54,55]. The fusion protein PML-RAR-enhances the expression of the S100 protein (a member of the S100 family of calcium-binding proteins), which in turn forms a heterotetrameric complex with annexin A2, a protein receptor with a strong affinity for plasminogen and t-PA as well as a potent cofactor for the conversion of plasminogen to plasmin. Thus, the surface-bound S100-annexin A2 complex promotes plasminogen activation and, at the same time, protects plasmin from inhibition [56]. Notably, the expression of annexin A2 can also be directly enhanced by PML-RAR-α [57].

From a clinical point of view, the severe hemorrhagic diathesis observed in patients with APL is the result of decreased clotting factors due to the activation of coagulation driven by the expression of high levels of tissue factor and cancer procoagulant by leukemic promyelocytes [54] and increased fibrinolysis. As a result of this double disease mechanism, the typical laboratory findings of APL are represented by a marked decrease in fibrinogen levels in combination with elevated FDPs.

More than 30 years ago, the introduction of all-trans retinoic acid (ATRA) for the APL treatment greatly reduced early hemorrhagic deaths in newly diagnosed patients [50]. By inducing the differentiation of leukemic blasts, ATRA is able to down-regulate S100 and annexin A2 overexpression, thus suppressing plasmin generation on APL cell surfaces and contributing to the correction of the hemostatic defect [51]. Given the central role of hyperfibrinolysis in the pathogenesis of bleeding in APL, a beneficial effect from the use of antifibrinolytic agents should be expected [58], but literature data are scanty and quite discordant [59]. The largest retrospective study of antifibrinolytics in APL demonstrated no improvement in transfusion requirements or major or fatal bleeding [60]. On the other hand, small case series on their use in APL have demonstrated some degree of improvements in laboratory markers of fibrinolysis and fewer transfusion requirements [61,62]. However, a few studies have raised some concerns regarding safety issues of these drugs in APL, particularly pertaining to thrombotic risk [63,64]. Further studies are therefore needed to retrieve more data on the safety and efficacy of fibrinolytic inhibitors for anti-hemorrhagic treatment in APL.

### 4.3. Trauma

Trauma is a leading global health burden, being responsible for over 6 million deaths worldwide each year [65]. Notably, nearly 50% of trauma deaths result from bleeding complications and occur in the first few hours after trauma [65].

Hemostatic abnormalities are a common finding in patients with trauma, being present in up to 25% of severely injured patients on arrival to the emergency department [65,66,67,68]. This early coagulopathy, called acute traumatic coagulopathy, is an endogenous process that develops on the combination of endothelial hypoperfusion and tissue injury, increased thrombin generation, platelet dysfunction, primary hyperfibrinolysis and fibrinogen depletion as key components [69,70,71,72]. There are several lines of evidence documenting a central role for accelerated fibrinolysis in the pathophysiology of acute traumatic coagulopathy [6]. Schochl and colleagues [73] reported a mortality rate of approximately 88% in trauma patients when hyperfibrinolysis occurred, detected by viscoelastic testing upon admission to the emergency department. Raza and colleagues [74], who assessed fibrinolysis activation through thromboelastometry and other assays (plasmin-antiplasmin complex and D-dimer levels), showed that primary hyperfibrinolysis occurred in the majority of trauma patients, with a degree of severity positively associated with significantly worse outcomes (transfusion requirements, morbidity and mortality).

**Figure 2:**
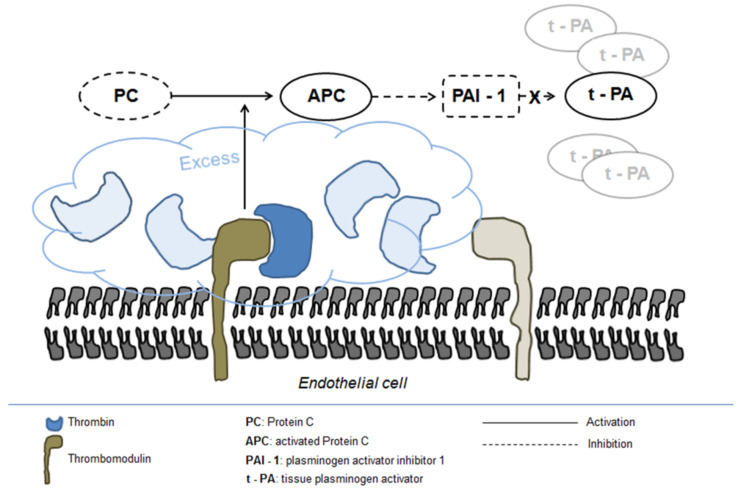
Fibrinolysis activation in severe trauma.

The precise mechanism underlying fibrinolysis activation in severe trauma is not completely elucidated, although the disruption of fibrinogen metabolism induced by hypothermia and metabolic acidosis and the resulting direct fibrinogenolysis have been indicated as potential mechanisms [68]. In addition, the activation of the protein C pathway seems to play a central role in enhancing systemic fibrinolysis and inducing fibrinogen depletion [65]. Protein C activation is markedly enhanced following trauma owing to the massive generation of thrombin and its complex formation with thrombomodulin on the endothelial surface [68]. Excess activated protein C in turn inhibits PAI-1 release leading to increased concentration of tPA (Figure 2) [72].

Several studies examined the therapeutic role of antifibrinolytic agents, in particular TXA, to reverse hyperfibrinolysis in severe trauma patients [75,76,77]. The largest body of evidence stems from the Clinical Randomization of an Antifibrinolytic in Significant Haemorrhage-2 (CRASH-2) trial [78], that enrolled 20,211 severely injured adult patients who were randomly assigned to receive TXA (loading bolus dose of 1 g and then infusion of 1 g over 8 h) or placebo within 8 h from trauma. All-cause mortality was significantly lower in the treatment group versus placebo group (14.5% versus 16%; relative risk (RR) 0.91, 95% CI 0.85–0.97; *p* = 0.0035). Similarly, the rate of deaths attributed to bleeding was reduced from 5.7% to 4.9% (*p* = 0.0077). A further sub-analysis of the CRASH-2 data revealed that the survival benefit of TXA only occurred when the drug was administered within 3 h after trauma (RR 0.79, 95% CI 0.64–0.97; *p* = 0.03) [79]. The Cochrane systematic review on antifibrinolytic drugs for acute traumatic injury found that, in the frame of the analysis of four trials enrolling 20,548 patients, TXA reduced the risk of death by 10% (RR 0.90, 95% CI 0.85–0.96; *p* = 0.002) without increasing the risk of adverse events [76]. TXA has also been extensively evaluated in the setting of traumatic brain injury. The recently published CRASH-3 trial enrolled 12,737 patients with isolated acute traumatic brain injury who were randomly assigned to receive TXA (loading dose 1 g over 10 min then infusion of 1 g over 8 h) or placebo [80]. TXA decreased the risk of head-injury-related deaths in patients with mild- to moderate head injury (RR 0.78, 95% CI 0.64–0.95). As in CRASH-2, early treatment was more effective than delayed treatment in patients with mild and moderate head injury (*p* = 0.005), with a 10% decrease in treatment effectiveness for every 20 min delay. Finally, a meta-analysis of 6 RCTs regarding the effect of TXA for traumatic brain injury compared with placebo showed that this antifibrinolytic agent was associated with lower mortality (RR 0.91, 95% CI 0.85–0.97; *p* = 0.0004) [81].

### 4.4. Post-Partum Hemorrhage

Post-partum hemorrhage, a major cause of maternal death worldwide, is commonly defined as blood loss of 500 mL or more within 24 h and is characterized by the early onset of hyperfibrinolysis [82,83]. The pathogenesis of increased fibrinolytic activity is poorly understood, but it seems to be similar (i.e., increased endothelial t-PA production and inhibition of PAI-1 via protein C activation) to that described in severe trauma [84]. In physiological pregnancies, FPD levels raise in the hours following delivery, but this increase is many times higher in women with post-partum hemorrhage [83]. The significant role played by fibrinolysis in post-partum hemorrhage is further supported by the recent finding of increased D-dimer levels and plasmin-antiplasmin complexes in the immediate post-partum period [85].

With this background, the clinical use of the antifibrinolytic agent TXA for the treatment of post-partum hemorrhage is appealing [86]. In the randomized, placebo-controlled trial (WOMAN, World Maternal ANtifibrinolytic), 20,060 women with post-partum hemorrhage following vaginal or cesarean delivery were assigned to receive either TXA (1 g TXA intravenously as soon as possible, followed by a further 1 g of TXA if bleeding continued after 30 min or restarted within 24 h of the initial dose) or placebo [87]. TXA reduced deaths due to bleeding (RR 0.81, 95% CI 0.65–1.00; *p* = 0.045) without adverse effects, especially when given as early as possible after the onset of bleeding. The survival benefit from the early administration of TXA was also documented by a recent meta-analysis of randomized trials involving more than 40,000 patients with severe acute hemorrhage (traumatic and post-partum hemorrhage) [88]. A possible explanation for the beneficial effect of early TXA administration in post-partum hemorrhage, as well in the trauma setting, may be the protection of residual fibrinogen stores (already consumed by the underlying bleeding condition) from a further hyperfibrinolysis-related decrease, thus maintaining the capacity to form a stable clot [6].

## 5. Conclusions

The goal of this narrative review is that of improving the awareness and the level of knowledge of physicians about the hemorrhagic disorders of primary fibrinolysis to speed up diagnoses and optimize the management of affected patients.

Primary hyperfibrinolysis is nowadays recognized as a distinct clinical bleeding entity associated with a variety of inherited and acquired disorders. Inherited bleeding disorders characterized by primary hyperfibrinolysis are a group of rare heterogeneous diseases, and their identification represents a diagnostic challenge that requires, besides a high-level clinical knowledge by physicians of these neglected diseases, the presence of specialized second-level hemostasis laboratories. It is therefore essential that patients with negative first-level coagulation screening tests but a clear and unequivocal positive personal and family history of bleeding are promptly deferred to specialized treatment centers for a proper diagnosis and treatment, which consists in most cases in the use of antifibrinolytics.

Similarly, the recognition of the role of primary hyperfibrinolysis in the pathogenesis of bleeding in some acquired and frequent conditions such as trauma and post-partum hemorrhage is very important because it helps to drive the most appropriate treatment. As the literature data support the beneficial effect of tranexamic acid when given early after bleeding onset in such conditions, the prompt identification of an associated hyperfibrinolytic state is essential to improve patients’ outcomes.

## Figures and Tables

**Table 1:** Summary of congenital and acquired hemorrhagic disorders of primary fibrinolysis.

Disorders of Primary Fibrinolysis	Type of Disorder	Laboratory, Clinical Characteristics and Therapy
Inherited	α_2_-antiplasmin deficiency	- Autosomal recessive, chromosome 17 - Type I quantitative deficiency, type II qualitative deficiency - Normal coagulation screening tests, ↓ ECLT - Bleeding diathesis in homozygotes, following trauma or surgery in heterozygotes - Treatment: TXA, FFP
	Plasminogen activator inhibitor-1 (PAI-1) deficiency	- Autosomal recessive, chromosome 7 - Quantitative and qualitative defects - Normal coagulation screening tests; ↓ ECLT - Bleeding following trauma or surgery in homozygotes; heterozygotes are asymptomatic - Treatment: TXA
	Quebec platelet disorder	- Autosomal dominant, chromosome 10 - Increase of u-PA platelet content - Bleeding following trauma or surgery, easy bruising, joint bleeds, hematuria - Treatment: TXA
Acquired	Liver cirrhosis	- Hyperfibrinolysis due to ↑ t-PA and ↓ TAFI; PAI-1 and α_2_-antiplasmin - Accelerated fibrinolysis during anhepatic phase of OLT - Mucosal and gastrointestinal bleeding - Treatment: TXA, aprotinin
	Acute promyelocytic leukemia	- APL cell-induced hyperfibrinolysis - ↓ fibrinogen and ↑ FDPs levels - Severe bleeding diathesis - Treatment: ATRA
	Trauma	- Hyperfibrinolysis associated with tissue injury and endothelial hypoperfusion - APC-mediated ↑ t-PA and ↓ PAI-1 - Treatment: TXA
	PPH	- Early onset of hyperfibrinolysis - Treatment: TXA

Abbreviations: ECLT, euglobulin clot lysis time; TXA, tranexamic acid; FFP, fresh frozen plasma; u-PA, urokinase plasminogen activator; t-PA, tissue plasminogen activator; PAI-1, plasminogen activator inhibitor 1; TAFI, thrombin-activatable fibrinolysis inhibitor; OLT, orthotopic liver transplantation; APL, acute promyelocytic leukemia; FDPs, fibrin degradation products; ATRA, all-trans-retinoic acid; APC, activated protein C; PPH, post-partum hemorrhage; ↑: increased; ↓: decreased.
